# cGMP Signaling in the Neurovascular Unit—Implications for Retinal Ganglion Cell Survival in Glaucoma

**DOI:** 10.3390/biom12111671

**Published:** 2022-11-11

**Authors:** Ameer A. Haider, Tonia S. Rex, Lauren K. Wareham

**Affiliations:** Vanderbilt Eye Institute, Department of Ophthalmology and Visual Sciences, Vanderbilt University Medical Center, Nashville, TN 37212, USA

**Keywords:** glaucoma, neurovascular coupling, neurodegeneration, glia, endothelial cell, retina, neurovascular unit

## Abstract

Glaucoma is a progressive age-related disease of the visual system and the leading cause of irreversible blindness worldwide. Currently, intraocular pressure (IOP) is the only modifiable risk factor for the disease, but even as IOP is lowered, the pathology of the disease often progresses. Hence, effective clinical targets for the treatment of glaucoma remain elusive. Glaucoma shares comorbidities with a multitude of vascular diseases, and evidence in humans and animal models demonstrates an association between vascular dysfunction of the retina and glaucoma pathology. Integral to the survival of retinal ganglion cells (RGCs) is functional neurovascular coupling (NVC), providing RGCs with metabolic support in response to neuronal activity. NVC is mediated by cells of the neurovascular unit (NVU), which include vascular cells, glial cells, and neurons. Nitric oxide-cyclic guanosine monophosphate (NO-cGMP) signaling is a prime mediator of NVC between endothelial cells and neurons, but emerging evidence suggests that cGMP signaling is also important in the physiology of other cells of the NVU. NO-cGMP signaling has been implicated in glaucomatous neurodegeneration in humans and mice. In this review, we explore the role of cGMP signaling in the different cell types of the NVU and investigate the potential links between cGMP signaling, breakdown of neurovascular function, and glaucoma pathology.

## 1. Introduction

Glaucoma is the leading cause of irreversible blindness worldwide, with global prevalence of the disease estimated to surpass 100 million cases by 2040 [1,2]. Glaucoma describes an umbrella of optic neuropathies that lead to the progressive degeneration of retinal ganglion cells (RGCs) and their axons that form the optic projection [3]. The disease is characterized by the increased sensitivity of RGCs and their axons to intraocular pressure (IOP), with degeneration of RGCs occurring sectorially over time; i.e., from one retinotopic sector to the next [4,5]. Current therapies aim to lower IOP using topical medication or surgical intervention; however, this is not effective for all patients and many progress to permanent vision loss. Moreover, a proportion of patients are normotensive, with IOPs within the typical range, and RGC axon degeneration still persists [6]. These observations suggest that the etiology of the disease is much more complex and alternative mechanisms of pathophysiology warrant further investigation.

Glaucoma shares comorbidities with a number of systemic vascular diseases, including diastolic hypotension, diabetes, ischemic cardiac disease, peripheral vasospasm, and arterial hypertension [7,8]. Advances in vascular imaging technology have provided evidence of a link between vascular dysfunction and glaucoma in both animal models and in the clinic [9]. In normotensive glaucoma patients, vascular changes in the retina occur, the most prominent being obstruction of ocular blood flow, resulting in glaucomatous optic neuropathy [10]. Adverse changes in blood vessel morphology that result in impaired ocular blood flow are also observed in glaucomatous retinas, irrespective of IOP level [9,10,11]. Nitric oxide-cyclic guanosine monophosphate (NO-cGMP) signaling is a primary mediator of vascular responses and has been implicated in glaucoma pathophysiology. In this review, we explore the role of cGMP signaling within the cells of the NVU and emphasize how dysfunctional cGMP signaling may lead to the breakdown of NVC evident in glaucoma. Using online literature databases such as PubMed and Web of Science, we searched combinations of terms such as “cGMP signaling”, “neurovascular”, “retina”, “retinal ganglion cell”, and “glia”, to conduct a comprehensive literature search. Initial inclusion criteria for publication date included articles from 2015 to the current date. However, highly cited earlier publications were also included if deemed an original publication of research in the field. The review herein will increase understanding of the vascular mechanisms of glaucoma and the potential role of NO-cGMP signaling, to facilitate the development of novel treatments for the disease.

## 2. The Retinal Neurovascular Unit and Glaucoma

**Figure 1:**
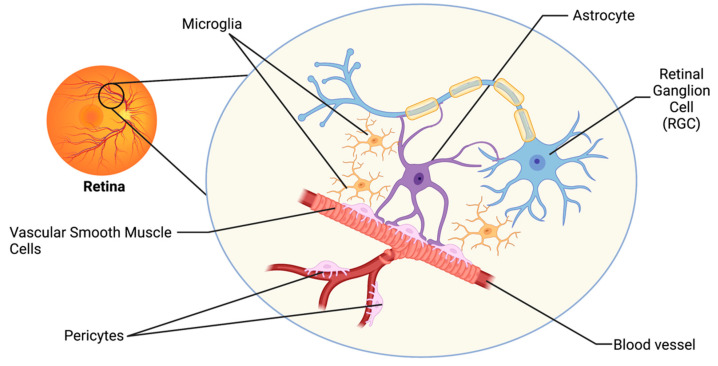
The retinal neurovascular unit. The retinal NVU consists of three main cell types: neurons (retinal ganglion cells; RGCs), glial cells (including astrocytes and microglia), and vascular cells (including endothelial cells, vascular smooth muscle cells, and pericytes).

The visual system has developed intricate regulatory mechanisms to fulfill the high metabolic demand of the retina. One such mechanism is neurovascular coupling (NVC), whereby an increased neuronal activity promotes signaling between cells of the neurovascular unit (NVU), resulting in increased supply of metabolites and removal of waste products by the vasculature [12]. The retinal NVU consists of three main cell types: neurons, glial cells (including astrocytes, microglia, and oligodendrocytes), and vascular cells (endothelial cells, vascular smooth muscle cells, and pericytes; Figure 1) [13]. Dysfunction in the NVU is evident in the pathophysiology of many neurodegenerative diseases of the central nervous system (CNS), including Alzheimer’s Disease and Parkinson’s Disease [3]. Such neurodegenerative diseases share many common pathophysiologies with glaucoma, and thus dysfunction in the NVU may also be a relevant mechanism of neurodegeneration of the visual system [3,13]. 

Vascular autoregulation and NVC mechanisms are severely compromised in glaucoma patients [14]. Dysfunction of the NVC response to light flicker was perturbed in patients with early-stage glaucoma progression, with only moderate IOP increases [14]. The direct effect of IOP on light-flicker-induced NVC was further tested using patients exposed to short-term induced IOP [15]; however, no changes in NVC response were observed. The change in NVC evident in early-stage glaucoma patients could be due to reduced function of RGCs or due to the dysfunction of cells in the NVU, which remains undetectable in the clinic. Rodent models of glaucoma have been a valuable resource in trying to better understand neurovascular mechanisms of the disease. In a murine model of elevated IOP, NVC responses were perturbed at the microvascular level and involved pericyte cell networks in the retina [16]. Specifically, elevation in IOP reduced pericyte-pericyte communication and perturbed NVC responses, suggesting that elevation in IOP directly impacts microcirculation in the retina [16]. Local reduction of microcirculation may have a profound impact on the health of RGCs across the retina. Detection of microcirculatory changes in humans, however, remains a challenge, due to the lack of equipment with the ability to image at microcapillary resolution. Animal models of glaucoma are therefore invaluable in identifying underlying mechanisms of NVC dysfunction and may be critical in exploring vascular dysfunction in glaucoma pathology. 

## 3. NO-cGMP Signaling in Glaucoma

The nitric oxide-cyclic guanosine monophosphate (NO-cGMP) signaling pathway is integral to NVC mechanisms and is the prime mediator of vasodilation in endothelial cells [9]. NO is produced by nitric oxide synthase enzymes in cells of the NVU in both physiological and pathophysiological conditions [17]. NO is lipophilic and can freely traverse cellular membranes, where it binds to its primary receptor, soluble guanylate cyclase (GC1; [18,19]). GC1 binding of NO stimulates the production of cGMP, a second messenger capable of binding to multiple cellular protein targets such as cyclic nucleotide phosphodiesterases (PDEs), cGMP-dependent protein kinases, and cGMP-gated ion channels [20,21,22,23]. cGMP signaling is multifaceted, with the potential to exert broad effects on cellular physiology, depending upon the cellular target that it binds.

Dysfunction in cGMP signaling has been implicated in glaucoma pathophysiology in human patients and in animal models of the disease. Levels of NO and cGMP in aqueous humor and plasma are reduced in glaucoma patients [24,25]. Furthermore, in a genome-wide association study (GWAS) of patients with primary open angle glaucoma (POAG), a single nucleotide polymorphism (rs11722059) located in the *GUCY1A3/GUCY1B3* gene locus was significantly associated with paracentral visual field loss in female glaucoma patients [26]. *GUCY1A3/GUCY1B3* encode the alpha and beta subunits of GC1, respectively [26]. Identification of a risk variant in the GC1 gene and expression of GC1 in human and rodent ocular tissues including RGCs [26] suggests that GC1 may have a role in the pathogenesis of glaucoma. 

The role of cGMP signaling in RGC physiology and pathophysiology has been demonstrated in animal studies. In a mouse model of induced ocular hypertension (i.e., the microbead model of occlusion [27]), increasing cGMP signaling through administration of tadalafil, a PDE inhibitor, prevented the degeneration of RGCs over time [28]. The effects of tadalafil on RGCs were IOP-independent; i.e., tadalafil did not reduce the elevated pressure of the model, and further in vitro experiments demonstrated that cGMP was able to reduce apoptotic signaling in RGCs [28]. Mice lacking the alpha catalytic subunit of guanylate cyclase (*GC1*^−/−^) were considered a novel murine model of POAG; the mice exhibit degeneration of RGCs with age, without large increases in IOP and an open irideocorneal angle [26]. *GC1*^−/−^ mice also develop retinal vascular dysfunction and aberrant retinal vascular morphology with age, and astrocyte morphology alterations precede vascular abnormalities [29]. These results suggest that NO-cGMP signaling functions to maintain homeostasis in cells of the NVU, and thus the NO-cGMP pathway remains an attractive area of investigation for glaucoma. Although activation of GC and increases in cGMP signaling have been successful in experimentally lowering IOP via interaction with the trabecular meshwork and ciliary muscles [30,31,32], lowering IOP is not always effective in preventing glaucoma pathology, especially in glaucoma patients with IOPs in the normal range. Understanding the potential roles of cGMP signaling within the NVU will give a better understanding of how cGMP signaling can be leveraged as a novel IOP-independent therapeutic for glaucoma. 

## 4. NO-cGMP Signaling in Vascular Cells

The vascular cells of the NVU in the brain consist of the endothelial cells, vascular smooth muscle cells, and pericytes. Nestled between the blood vessel lumen and the smooth muscle cells, the endothelium is responsible for modulation of vascular tone, thrombus formation, cell adhesion, smooth muscle proliferation, and sequestration of inflammatory factors [33]. Within the retinal NVU, the function is similar; endothelial cells line retinal vessels responsible for RGC blood supply and smooth muscle cells control vascular tone [9,34]. Critical to endothelial function is cGMP signaling; outside of the CNS, cGMP signaling plays a major role in decreasing endothelial cell permeability and is responsible for smooth muscle cell relaxation and subsequent vasodilation [35,36]. 

**Figure 2:**
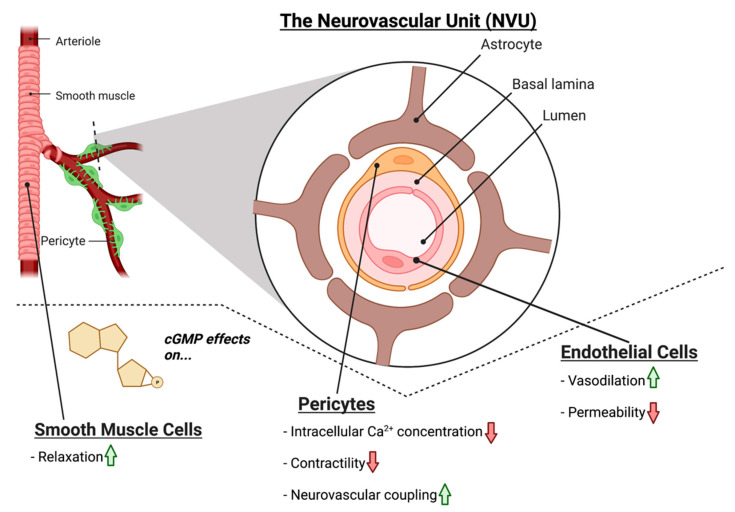
The effects of cGMP on vascular cells of the retinal NVU. cGMP increases smooth muscle cell relaxation. In pericytes, cGMP has been shown to reduce intracellular Ca^2+^, decrease contractility, and increase NVC mechanisms. In endothelial cells, cGMP has been shown to increase vasodilation, while reducing endothelial cell permeability.

Within the NVU, NO-cGMP signaling plays a similar role in modulating vascular tone; NO produced in the endothelium acts on GC1 in smooth muscle cells, to induce vasodilation via smooth muscle relaxation (Figure 2) [34]. Moreover, cGMP is also implicated in decreasing the permeability of the endothelium of the NVU. Experimental treatment with the cGMP agonist 8-Br-cGMP decreased the permeability of an in vitro blood–brain barrier (BBB) model [37]. Inhibition of GC1 by ODQ (1H-[1,2,4]oxadiazolo[4,3-a]quinoxalin-1-one) increased permeability, and inhibition of the cGMP-dependent protein kinase prevented the cGMP-mediated decrease in permeability [37]. Hence, NO-cGMP signaling is critical to the vasodilation of blood vessels and in maintaining the integrity of the endothelial cell monolayer. Endothelial cell dysfunction is observed in patients with POAG [9,38,39,40,41]; in both normotensive glaucoma (NTG) and hypertensive glaucoma patients, impairment of endothelial-cell mediated vasodilation in response to intravenous acetylcholine was evident [42,43,44]. Taking into consideration the evident endothelial dysfunction in glaucoma and the role of NO-cGMP in endothelial permeability and function, it is possible that breakdown of NO-cGMP signaling may lead to dysfunction of endothelial cells, which may underlie vasculature-related glaucoma pathophysiology. Further studies are needed, to explore the potential link between dysfunctional NO-cGMP signaling in endothelium and glaucomatous degeneration.

The vascular cells of the NVU also include pericytes; however, exploration of their role within the retinal NVU in glaucoma pathology is in its infancy compared to other cells of the retinal vasculature [9]. Pericytes are situated in the basement membrane of microvessels and are responsible for a multitude of functions, including maintaining vascular stability, providing structural support for the blood vessels, mediating vasodilation and vasoconstriction, and BBB maintenance [13,45,46,47,48,49]. The tone of pericyte-containing microvessels is largely controlled by the balance between Ca^2+^-mediated contractility and NO-mediated relaxation [50]. Recently, a key role for pericytes in retinal NVC and glaucoma pathology was uncovered [16]. The identification of inter-pericyte tunneling nanotubes (IP-TNTs) between pericyte cells in the retina led to the discovery that pericytes use IP-TNTs to coordinate vascular responses to neuronal activity across the retina [51]. Damage to IP-TNTs in glaucoma impairs light-evoked neurovascular coupling via persistent Ca^2+^ influx, causing vasoconstriction [16]. Similarly to smooth muscle cells, pericytes contract and relax to control vessel tone; however, they are also directly influenced by oxygen tension in the retina; in ischemic conditions, they become dysfunctional and fail to relax after constriction, exacerbating the lack of blood supply [9,49,52,53]. Interestingly, in aged rat retinas, there is less pericyte-endothelial cell contact [54]. Since age is a prominent risk factor for glaucoma, reduced pericyte-endothelial cell contact may predispose aged retinas to glaucomatous damage through weakened NVC. There is increasing evidence that vascular supply of blood is impaired in glaucoma [55]. It is therefore plausible that glaucomatous RGC degeneration may be exacerbated by impaired pericyte activity, reducing blood supply and metabolic support to the cells [16].

The role of cGMP signaling in pericytes located outside of the CNS has been explored extensively and may provide a mechanistic link between retinal pericyte dysfunction (specifically pericyte-associated vasoconstriction and NVC breakdown) and the vascular-associated glaucomatous pathology observed in patients. In liver pericytes, exogenous NO supplementation and inhibition of cGMP breakdown via PDE inhibitor sildenafil citrate diminished the extent of endothelial dysfunction caused by contraction of active pericytes [56]. Moreover, in the mouse brain, the GC1 inhibitor ODQ blunts the NO-induced relaxation of pericytes, decreases pericyte Ca^2+^ levels, and decreases whisker-pad stimulation-induced vasodilator responses in capillaries [57]. This is consistent with the findings that pericytes mediate capillary dilation in the brain [58], and that pericyte-deficient mice exhibit neurovascular uncoupling, reduced oxygen supply to the brain, and metabolic stress [59]. In retinal pericytes, Ca^2+^ influx, which causes pericyte contraction, can be inhibited via 8-Br-cGMP (a cGMP agonist), as well as SNP (a NO donor) [60]. It is therefore likely that NO-cGMP-mediated control of pericyte Ca^2+^ is related to changes in pericyte contractility and may impact retinal microvascular function. Across the vascular cells of the NVU, breakdown of NO-cGMP signaling may contribute to dysfunction in endothelial and smooth muscle-cell mediated vasodilation, endothelial monolayer permeability, and pericyte vasoactivity, all of which are pertinent to the vascular component of glaucomatous damage (Figure 2). However, further studies must refine the link between defective NO-cGMP signaling in these cells and the alterations in NVC present in glaucoma.

## 5. NO-cGMP Signaling in Glial Cells

The retinal glia, including astrocytes, Müller glia, and microglia, were thought to be relatively passive elements in the CNS; however, recently, it has become clear that they play a critical intermediary role between the neurons and vascular cells of the NVU [13,61,62]. The predominant glial cells in the retina are the Müller glia (MG), which span the retinal layers, from the inner border with the vitreous humor, to the photoreceptor layer. Astrocytes are present in the superficial layer of the retina and make intimate connections between RGCs and blood vessels. Astrocytes and MG together form the blood–retinal barrier (BRB) and are integral to retinal NVC [13,63,64]. Astrocytes play a critical role in shuttling energy resources to neurons, responding to glutamatergic activity by producing lactate and releasing it into the extracellular space, which neurons then utilize for metabolic demands [65,66,67]. Astrocytes also regulate neurotransmitter release and re-uptake, to help maintain neuronal homeostasis [13,68,69,70,71]. In humans, regarding glial cells, the fovea contains only MG subtypes, indicating that these cells alone are sufficient to supply the metabolic needs of the RGCs [13,71]. Microglia are also resident glial cells that span multiple layers of the retina, yet their exact role in the basal NVU function of the retina is less understood compared to other glial cell types [72]. Recently, it has been shown in the brain that microglia directly and dynamically contact endothelial, smooth muscle cells, pericytes, and astrocytes, and are able to regulate cerebral blood flow via vasoactive agents, including NO and cGMP [73]. These results suggest that microglia, along with MG and astrocytes are important for NVC in the retina.

In glaucoma, glial dysfunction and/or activation may contribute to breakdown in NVC, rendering RGCs more susceptible to degeneration. Given their localization in the RGC layer and role in metabolic support, changes in astrocyte physiology may have a large impact on the health and physiology of RGCs. During pathological conditions in the retina, astrocytes respond robustly with gross morphological and physiological changes, which may prompt the dysfunction and degeneration of RGC axons [9,67,74,75]. Important to astrocytic cellular networks is the intercellular communication via connexin proteins, which form gap junctions between astrocytes and other cells of the NVU [9,67]. Changes in astrocyte morphology and connexin expression in the NVU have been demonstrated in early stages of glaucoma in animal models [9,76,77,78,79]. Furthermore, astrocytic connexin-43 (Cx43), the most abundant connexin in astrocytes, was shown to facilitate the movement of energetic resources in the optic projection in response to injury [80]. The role of MG and microglia in neurovascular function is less clear; however, MG undergo reactive gliosis in glaucoma, and increasing evidence supports a critical role of MG in maintenance of RGC physiology [81,82]. Finally, reactive microglia in glaucoma release pro-inflammatory cytokines, triggering RGC degradation [83].

Examining the role of NO-cGMP signaling in the NVU may help to elucidate the relationship between glia, vascular function, and glaucoma pathology. In the CNS, decreased GC1 expression is detected in the reactive glia of post-mortem patients with neurodegenerative diseases such Alzheimer’s disease and multiple sclerosis [84,85,86]. In cultured retinal MG, addition of cGMP analogues activates a calcium permeable, non-selective cation current via the opening of cGMP-gated channels, indicating that cGMP signaling, in part, regulates MG physiology [87]. In the microglia, cGMP signaling pathways are involved in inflammatory gene expression [84,88,89,90,91,92,93,94]. Pharmacological inhibition of GC1 prevents activation and migration of microglia to injured or inflamed sites in the CNS; recruitment can be rescued with the addition of a cGMP analog, suggesting that microglial recruitment is, at least in part, mediated by cGMP signaling [95,96].

**Figure 3:**
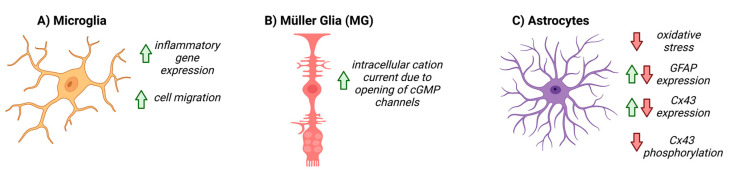
The known effects of cGMP on glial cells. Red arrows represent downregulation, or negative activity, green arrows represent increased expression, or positive activity. (**A**) cGMP upregulates inflammatory gene expression and cell migration in microglia, (**B**) increases cation current within Müller cells via opening of cGMP-gated channels, and (**C**) decreases oxidative stress, differentially affects GFAP and Cx43 expression, and decreases Cx43 phosphorylation in astrocytes.

To date, the role of NO-cGMP signaling in astrocyte function has been explored in many contexts, both within the CNS and systemically, and has been summarized in Figure 3. In cultured rat cerebellar astrocytes, cGMP directly affects the motility and morphology of cells and expression of glial fibrillary acidic protein (GFAP), suggesting that cGMP signaling directly modulates astrocyte physiology [97,98]. In focal brain injury, inhibiting PDE increased cGMP and altered the glial inflammatory response, decreased oxidative stress and cell death, and increased angiogenesis (Figure 3) [84]. A potential role for cGMP signaling in retinal astrocyte physiology has been reported in a study using a germline GC1 alpha subunit knockout mouse [29]. In the study, *GC1^−/−^* mice that developed RGC degeneration with age [26,28] were shown to have an altered retinal vascular phenotype; blood vessels in the peripheral retinal vasculature were dilated and exhibited shorter capillary branching [9,29]. Interestingly, vascular changes were preceded by an increase in glial fibrillary acidic protein (GFAP) expression in astrocytes and changes in astrocyte morphology, specifically astrocytic matting around blood vessels and enlarged bulbous astrocytic endfeet [29]. The effects in astrocytes may be due to reduced cGMP signaling in the astrocytes themselves; in human and rat cultured astrocytes, cGMP-PKG activity directly increases GFAP expression and RhoA inhibition, which are responsible for astrocyte morphological changes [97,99]. Moreover, changes in astrocyte connexin expression have also been observed in *GC1^−/−^* mice [29]. Cx43 is the most prevalent connexin in astrocytes, and *GC1^−/−^* mice exhibit a greater decrease in connexin-43 levels with age [29,67]. Connexins are crucial in astrocyte cell communication in the NVU, and increases in pressure in vitro cause a decrease in gap-junction communication via alterations in connexin activity [77]. cGMP/PKG activity has been shown to alter connexin activity, specifically through phosphorylation, hinting at a link between cGMP activity, connexins, and astrocyte function in the NVU [100]. Given the aforementioned link between connexin expression and glaucoma, and the effect of cGMP levels on astrocyte connexin physiology, cGMP could be relevant to the dysfunction of the NVU observed in glaucoma.

In cell types outside of the CNS, Cx43 expression and function is mediated by cGMP signaling. Rat mesangial cells treated with the NO donor S-nitroso-N-acetylpenicillamine (SNAP) or 8-Br-cGMP increased expression of Cx43 [101]. Additionally, *GC1*^−/−^ mice exhibit Cx43 phosphorylation in the intercalated disc of cardiac tissue, resulting in dysfunctional gap junction electrical conductivity [102]. Finally, in rodent vascular smooth muscular cells, Cx43 expression increases in response to either 8-Br-cGMP treatment or BAY 41-2272, a GC1 stimulator, and PKG inhibition reverses this increase [103]. The potential role of cGMP in astrocyte communication and gap junction function is intriguing; however, future investigations between NO-cGMP signaling and Cx43 expression in the retinal NVU are warranted.

Besides connexins, cGMP-gated channels also impart changes in astrocyte physiology that may perturb vascular function in glaucoma. cGMP-gated Ca^2+^ channels have been confirmed to populate the astrocytes of the rat forebrain, midbrain, and hindbrain, and are important for astrocyte function [104]. MG also likely possess cGMP-gated Ca^2+^ channels [87]. Within the endfeet of astrocytes in the NVU, Ca^2+^ signaling plays a critical role in maintaining the integrity of the BBB [105]; yet, it is unknown if the cGMP-gated Ca^2+^ channels specifically contribute to the abnormal vascular function of astrocytes in the glaucomatous NVU. cGMP-gated Ca^2+^ channels in astrocytes have been investigated as a potential therapeutic target for vascular dysfunction in Alzheimer’s disease [106]. Given that glaucoma shares many pathological commonalities with Alzheimer’s disease [3], investigating the putative role of cGMP-gated Ca^2+^ channels in retinal astrocytes in glaucoma may uncover a novel therapeutic target.

## 6. NO-cGMP Signaling in Retinal Neurons

In glaucoma, the degeneration of RGCs and their axons in the optic projection results in irreversible vision loss. The role of NO-cGMP signaling in RGCs appears to be multifaceted. In CNS neurons, NO-cGMP signaling is important in NVC, synaptic plasticity, and synaptic transmission [107]. GC1 is expressed in pre- and post-synaptic terminals and in neuronal soma and spines [107,108]. GC1 is also evident in RGC soma in the retina [26]. The depolarization of neuronal membranes stimulates Ca^2+^ influx into RGCs, which activates neuronal nitric oxide synthase, leading to NO production and release, stimulating vasodilation in its target vascular cells via cGMP [62]. Interestingly, *GC1^−/−^* mice [26] develop RGC degeneration with age, and increasing availability of cGMP has a neuroprotective effect in murine glaucoma models [28]. Furthermore, primary RGC cultures and mouse retinal explants exhibit decreased apoptotic signaling when supplemented with cGMP [109]. Within the neuron itself, cGMP and its downstream effector PKG are key mediators for the neuroprotective effects of NO [110], and multiple studies have confirmed the neuroprotective effect of stimulating the NO-cGMP-PKG pathway in RGCs [28,111,112,113] and in other neurons of the CNS [110,114]. However, it is worth noting that activation of PKG has shown neurotoxicity, rather than neuroprotective effects, in RGCs [115,116], suggesting that alternative downstream effectors of cGMP, such as cGMP-gated channels and transcription factors, such as cAMP response element binding protein (CREB), may underpin the neuroprotective capacity of cGMP. cGMP-gated channels have been identified in RGCs and are responsible for modulation of neuron excitability [117,118]. The transcription factor CREB is responsible for neuroprotective functions and is phosphorylated and activated by PKG in CNS neurons [110,119,120,121]. Alternatively, cGMP, via PKG, can also modulate intracellular calcium signaling in RGCs [122] and therefore may be responsible for limiting the neurotoxic effects to neurons of high intracellular Ca^2+^ levels. Since cGMP has multiple downstream effectors that may contribute to the neuroprotective effects observed in RGCs, further research is needed to distinguish the apparent neuroprotective and neurodegenerative arms of the cGMP signaling cascade.

## 7. Conclusions and Future Directions

Glaucoma is a devastating optic neuropathy afflicting millions worldwide, and yet the complex etiology of the disease results in the ensuing neurodegeneration not receiving effective treatment for many individuals. While the past understanding and treatment of the disease has focused on controlling IOP, the only current modifiable risk factor, recent research has elucidated a substantial contribution of retinal vasculature dysfunction to disease progression [3,7,9,34]. Whether vascular dysfunction occurs early in glaucoma triggering neurodegenerative events, or whether RGC degeneration leads to the disruption of NVC remains challenging to determine.

It is clear that NO-cGMP signaling is integral to NVC between retinal neurons and the retinal vasculature and that proper cGMP signaling helps to maintain the integrity of the BRB. Interestingly, NO-cGMP signaling has additional roles in glial cell physiology and homeostasis. While we have highlighted the importance of NO-cGMP signaling in multiple cell types of the NVU and have provided evidence of dysfunctional cGMP signaling in glaucoma, a number of questions remain. Specifically, in glaucoma, how might dysfunctional NO-cGMP signaling in cells of the NVU directly contribute to RGC degeneration? Given the ubiquity of cGMP in cells of the NVU, answering this question will not be facile. Future studies implementing cell-specific knockouts or knockdowns of GC1, combined with animal models of neurodegeneration will be critical in elucidating the relationship between NO-cGMP signaling in the NVU, vascular dysfunction, and glaucoma pathology. Disentangling the specific role of NO-cGMP in cells of the NVU and the relationship to glaucomatous degradation would be indispensable in providing a more comprehensive understanding of the vascular mechanisms of the disease and help to provide effective novel therapeutic targets.
